# Supplementary material for: Wireless multi-lateral optofluidic microsystems for real-time programmable optogenetics and photopharmacology
Source: Nat Commun. 2022 Sep 22;13:5571. doi: 10.1038/s41467-022-32947-0 (PMC9500026; doi:10.1038/s41467-022-32947-0)
Supplement: Supplementary file 1 — Supplementary Information [file 41467_2022_32947_MOESM1_ESM.pdf]

Supplementary Information

Wireless multi-lateral optofluidic microsystems for real-time programmable optogenetics and photopharmacology

Supplementary Table 1. Comparison of various state-of-art wireless optofluidic systems.

	Water electrolysis			Thermal-activated		
	Wireless multilateral platform (Wu et al, this paper)	Battery-free wireless optofluidic cuff (Zhang et al., 2019)	Battery-free wireless optofluidic probe (Zhang et al., 2019)	Battery-powered, smartphone-controlled device with replaceable cartridges (Qazi et al., 2019)	Battery-free, ultra-high-frequency RF wireless device (Noh et al., 2018)	Battery-powered infrared-controlled devices (Jeong et al., 2015)
Weight	0.15 g	0.29 g	0.3 g	2 g	0.22 g	1.8 g
Number of probes	1 or 2	1	1	1	1	1
Number of drug and light channels	2 drug reservoirs & 2 LEDs	4 drug reservoirs & 1 LED	4 drug reservoirs & 1 LED	4 drug reservoirs & 2 LEDs	1 drug reservoir & 1 LED	4 drug reservoirs & 1 LED
Temperature increase in reservoirs	< 0.2 ^{o}C	< 0.2 ^{o}C	< 0.2 ^{o}C	Up to 60 ^{o}C	Up to 60 ^{o}C	Up to 60 ^{o}C
µ-pump Power consumption	<1 mW	<1 mW	<1 mW	>100 mW	>100 mW	>100 mW
Operation (Frequency)	Radio frequency (13.56 MHz)	Radio frequency (13.56 MHz)	Radio frequency (13.56 MHz)	Bluetooth (2.45 GHz)	Resonant frequency (1.8-3.2 GHz)	Infrared (38 kHz)
Real-time programmability	Yes	No	No	Yes	No	No
Control range	25 cm, extendable without limit with additional transmission antenna	25 cm, extendable without limit with additional transmission antenna	25 cm, extendable without limit with additional transmission antenna	10-100 m	~ 10 cm, extendable without limit with additional transmission antenna	~ 2 m
Multiple Independently controllable animals within a large group	Yes (Control up to 256 devices in a single field)	No	No	Yes (Control up to 7 active devices at a time)	No	No
Flow rate modulation	Yes, real-time modulation	Yes, limited, need to preprogram	Yes, limited, need to preprogram	No	No	No
Leakage/backflow prevention	Check valve prevents potential leakage and backflow	No	Gold film to avoid leakage	No	No	No
Refillability	Yes	Yes	Yes	Yes, by replacing cartridges	No	No
Full implantability	Yes	Yes	Yes	No	Yes	No

Supplementary Table 2. Materials and process pricing for wireless optofluidic systems.

Parts	Bulk cost	Individual device cost	Notes
PCB Foundry outsource	$7,550/500 devices	$15.10	
PCB Foundry outsource	$127/40 probes	$3.18	
Components Digikey		$2.66	Sum of 6 components
Parylene coating done by SCS	$37.50/20 devices on a panel, 20 panels	$1.88	
Polyimide tape to cover electrodes and probe pads	$15/roll (36yd)	$0.02	~1" per device
COC for pump chamber	$25/153 chambers	$0.16	Assuming 100% yield
Marine epoxy	$12/1 fl oz	$0.60	
SIS (Polystyrene-block-isoprene-block-polystyrene) powder from Sigma-Aldrich for flexible membrane	$88/250grams		1 grams/5000
Toluene for flexible membrane	$46.60/100mL		10 ml/5000
Gold for flexible membrane			
Titanium for flexible membrane			
Si02 for flexible membrane	11.25/5000	$0.003	Ebeam deposition
COC for drug reservoir	$25/176 reservoirs	$0.14	Assuming 100% yield
2 silicone wafers - fluidic channel mold	N/A	negligible	Reusable
SU8-2025 photoresist - fluidic channel mold	N/A	negligible	Reusable
PDMS - fluidic channel; cover for components	$32/453g	$0.21	~30g used to spin coat two wafers
Adhesive - bonding		$1.05	cost is unknown
Scotch tape - top port seal	$1.59/700"	$0.01	~1" per device
GRAND TOTAL		$25.01	

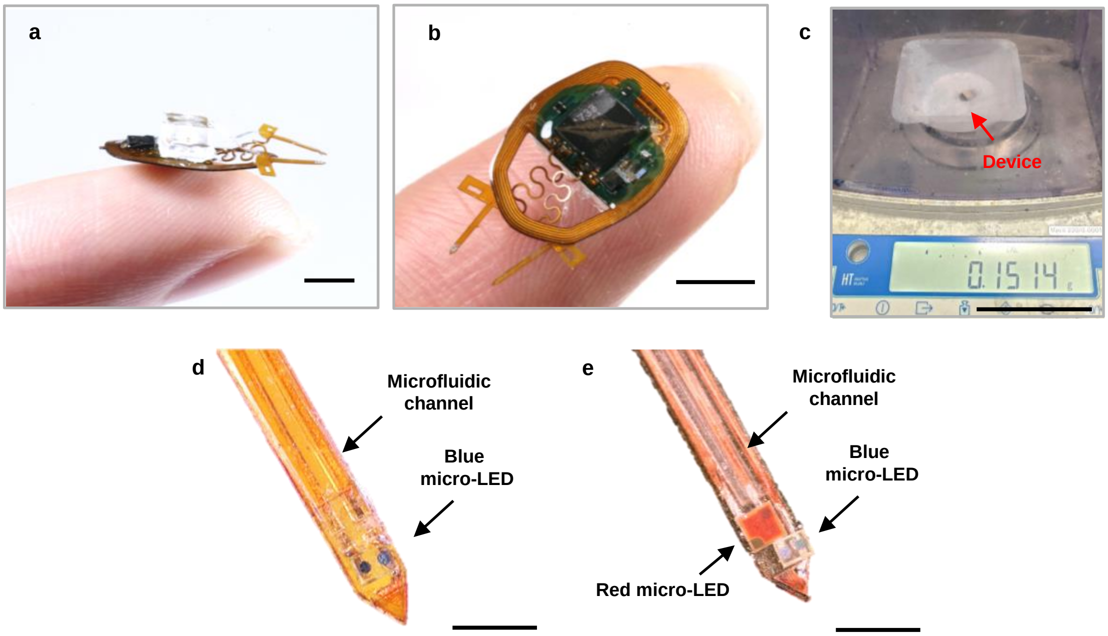

Supplementary Figure 1. Device closed-up photos and weight. (**a**) Optical image of the side view of a bilateral optofluidic device. Scale bar: 5 mm. (**b**) Optical image of the back view of a bilateral optofluidic device. Scale bar: 5 mm. (**c**) The weight of a bilateral optofluidic device is ~0.151g. Scale bar: 10 cm.(**d**) Close-up image of a bilateral probe with a single µ-fluidic channel for pharmacology and a blue µ-LED for optical stimulation. Scale bar: 1 mm. (**e**) Close-up image of a bi-channel probe with a dual µ-fluidic channels for pharmacology and a set of one blue µ-LED and one red µ-LED for optical stimulation. Scale bar: 1 mm.

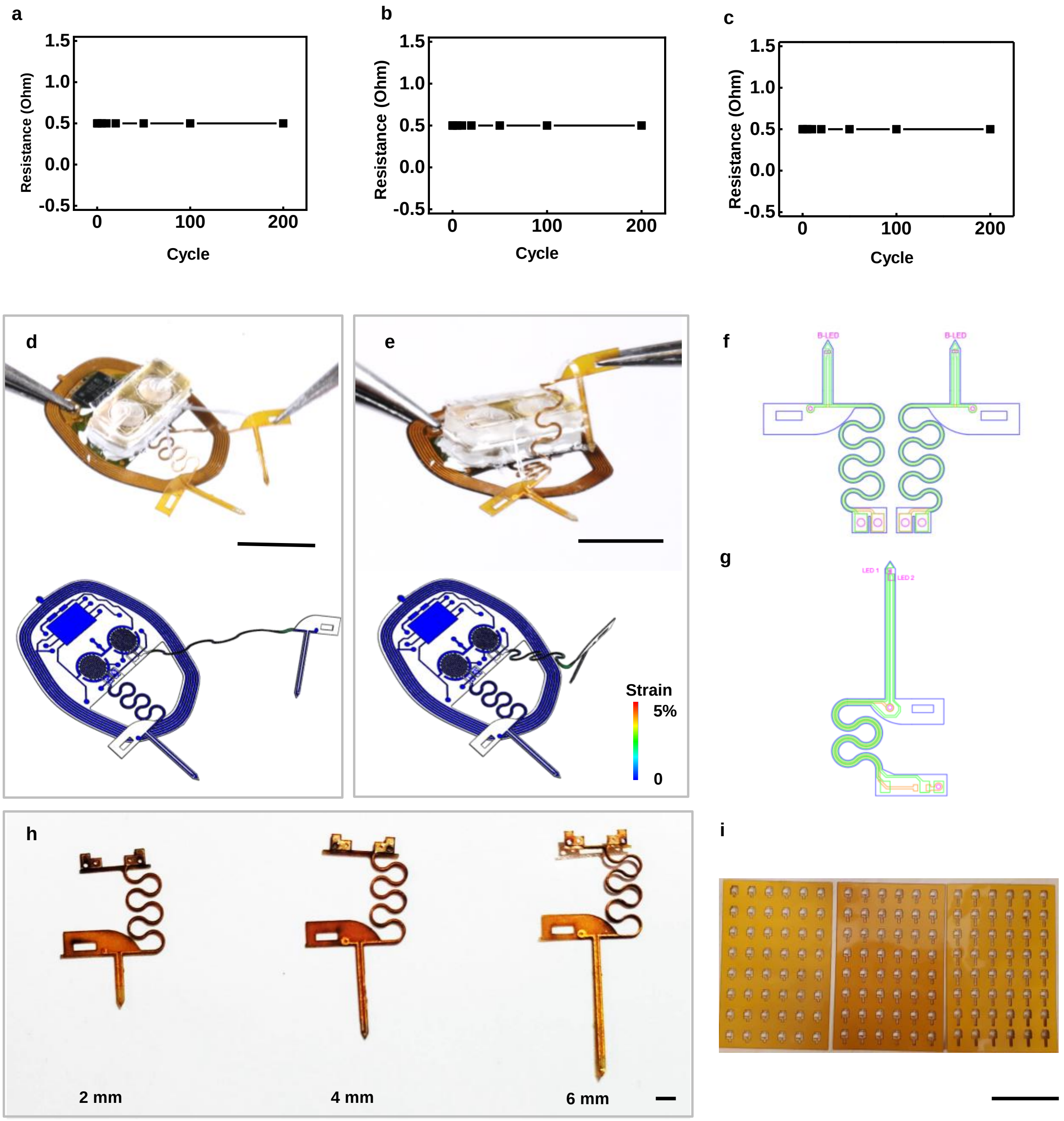

Supplementary Figure 2. Mechanical properties of serpentine optofluidic probes. (**a**)-(**c**)**.** Failure tests of serpentine under tensile strain 50% (4 mm to 6 mm) (**a**), tensile strain 100% (4 mm to 8 mm) (**b**) and compression strain 50% (4 mm to 2 mm) (**c**), 1Hz. (**d**) and (**e**) Photos and mechanical modeling of device probe under stretching and bending during the implantation process. Scale bar: 5 mm. (**f**) and (**g**) CAD design of bilateral and bi-channel tips. (**h**) Customizable probes to target different brain regions. Scale bar: 1 mm. (**i**) Panels of optofluidic probes with different length. Scale bar: 5 cm. Source data are provided as a Source Data file.

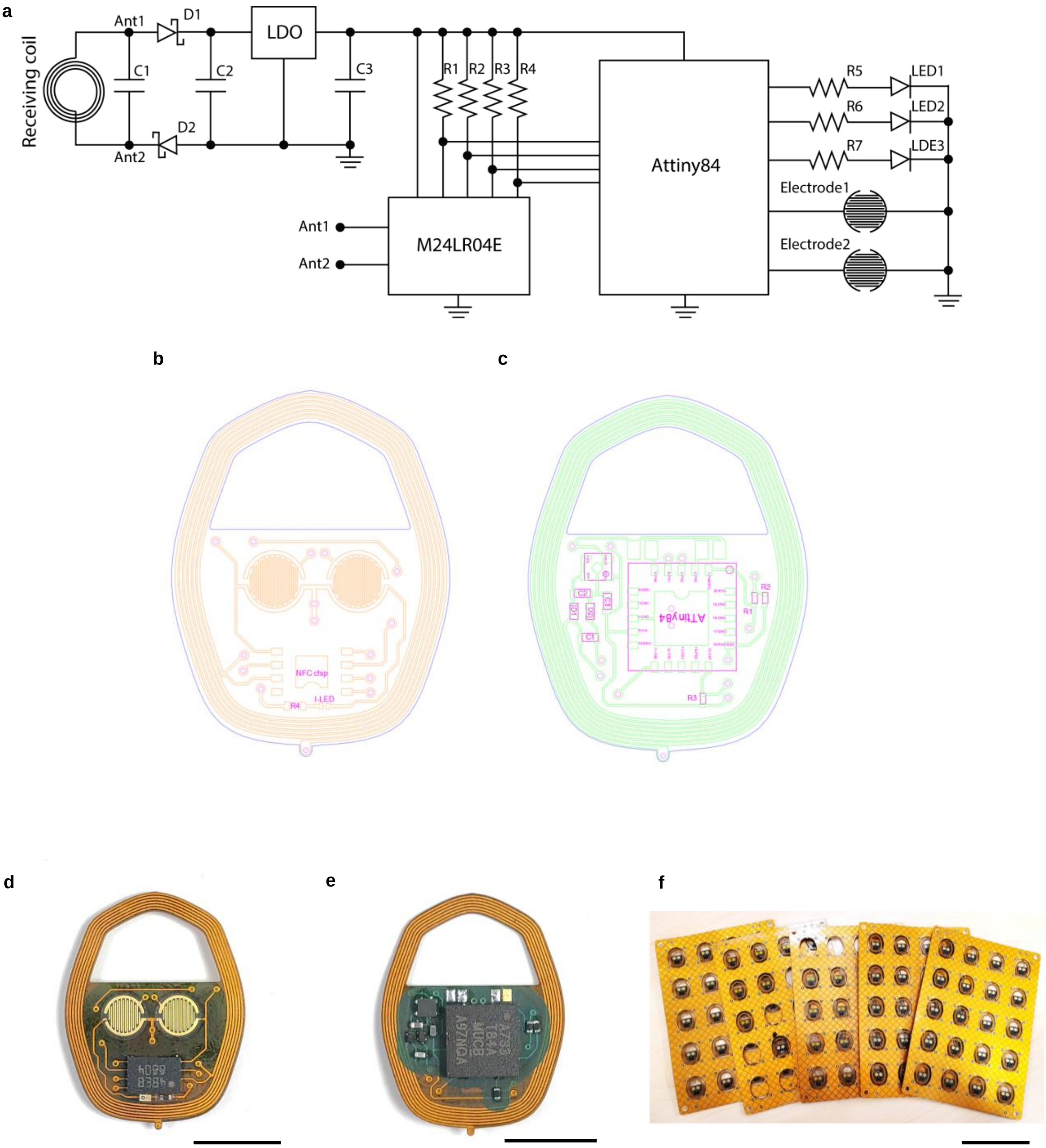

Supplementary Figure 3. Wireless optofluidic electronic circuitry design. (**a**) Circuit diagram. (**b**) and (**c**) Electronic circuitry CAD design. (**d**) and (**e**) Optical images of the top and bottom vies of the electronic circuitry with all the components. Scale bar: 5 mm. (**f**) Panels of electronics. Scale bar: 5 cm.

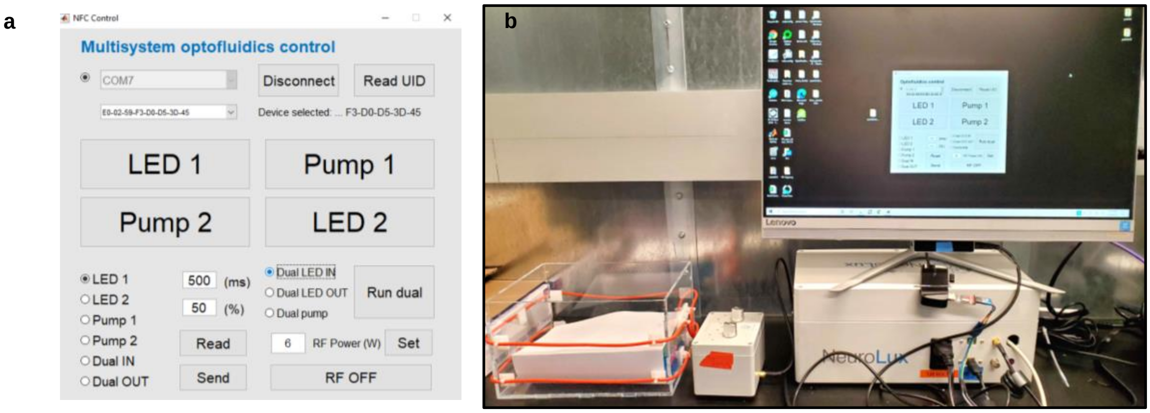

Supplementary Figure 4. GUI, powering and communication system of wireless optofluidic system. (**a**) Graphic user interface of the bilateral optofluidic system. (**b**) Power transmitting and controlling system.

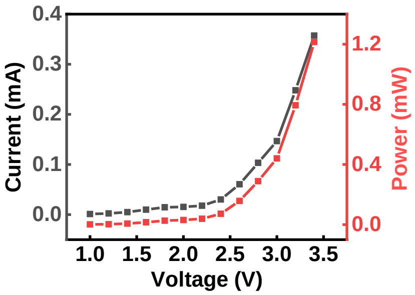

Supplementary Figure 5. I-V curve and power consumption of the electrochemical pump. Source data are provided as a Source Data file.

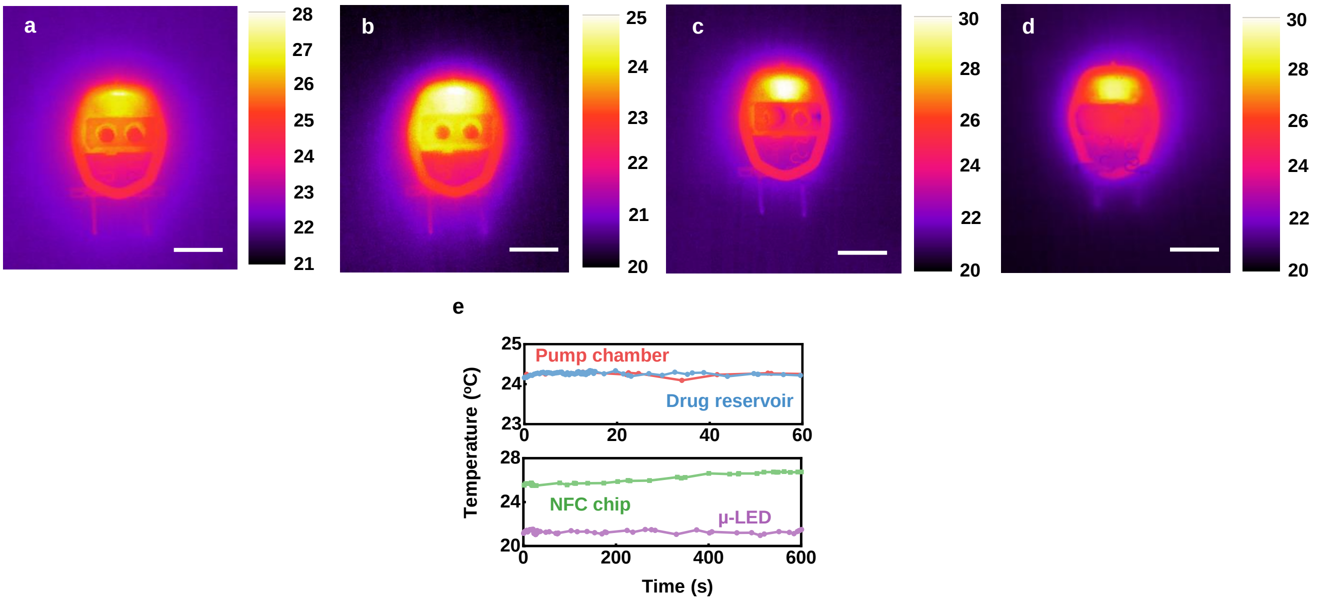

Supplementary Figure 6. Thermal characterization of µ-pump. (**a**)-(**d**) Infrared images of NFC chip (**a**), µ-LED (Frequency: 200ms, Duty cycle: 5%), pump chamber (**c**) and drug reservoir (**d**). Scale bar: 5 mm. **e.** Temperature change of pump chamber and drug reservoir during 60 second operation (frequency: 200ms; duty cycle: 50%) as well as electronics (NFC chip as representative) and µ-LED during 10mins operation (frequency: 200ms; duty cycle: 5%). Source data are provided as a Source Data file.

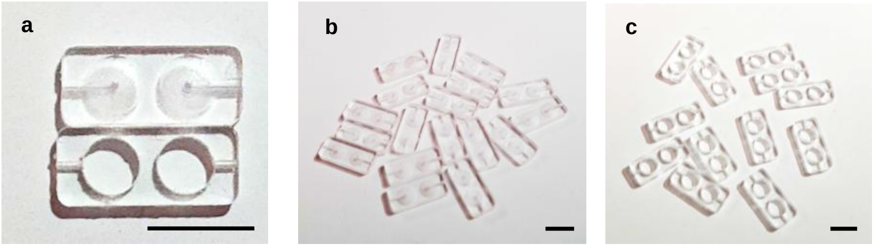

Supplementary Figure 7. Photos of µ-pump drug reservoirs and pump chamber. (**a**) Closed-up photo of µ-pump drug reservoirs and pump chamber. Scale bar: 1 cm. (**b**) - (**c**) Mass-producible drug reservoirs and pump chamber fabricated by milling machine. Scale bar: 1 cm.

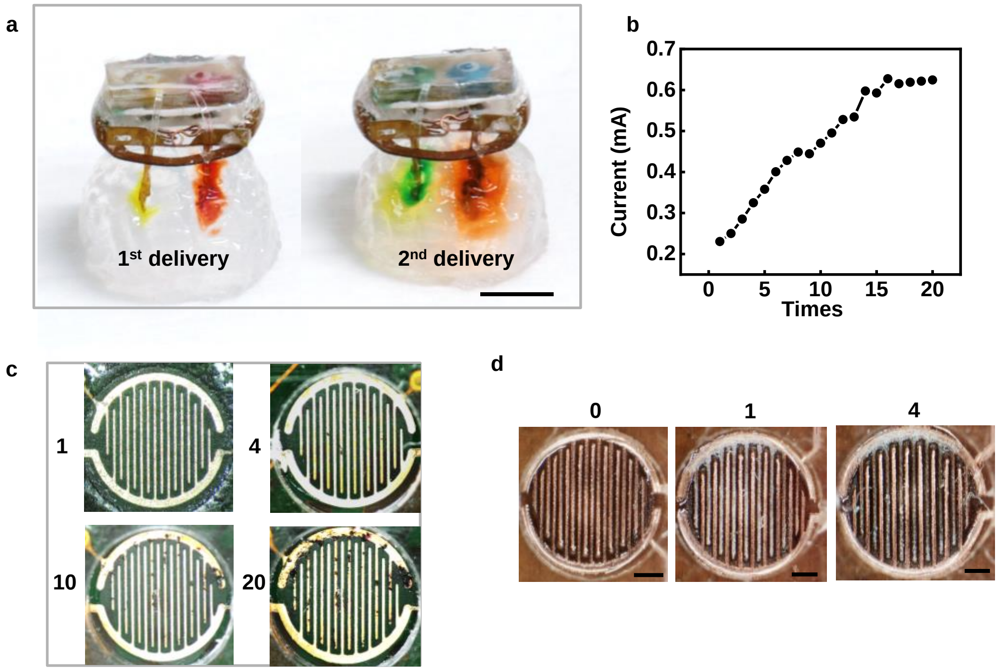

Supplementary Figure 8. Refillability of electrochemical μ-pump. (**a**) Hydrogel illustration of the refillability and repeatability of the optofluidic device (frequency: 4Hz, duty cycle: 100%). Scale bar: 5 mm. (**b**) Effective current change with times of usage**.** (**c**) Optical images of the electrochemical electrodes after 1, 4, 10 and 20 times of usages (frequency: 4Hz, duty cycle: 50%, time: 2 min). (**d**) Optical images of copper electrochemical electrodes without gold coating after 0, 1, 4 cycles of use in electrochemical pumping. Scale bar: 0.5 mm (frequency: 4Hz, duty cycle: 50%, time: 2 min). Source data are provided as a Source Data file.

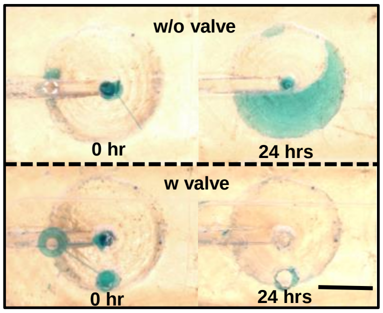

Supplementary Figure 9. Microscopic images of the drug reservoir without and with passive check valve at 0 and 24hrs after delivery. The microfluidic channels were immersed in a blue dyed water. The device without valve shows a retraction of fluid from outlet while the device with valve did not have any fluid retraction. Scale bar: 1 mm

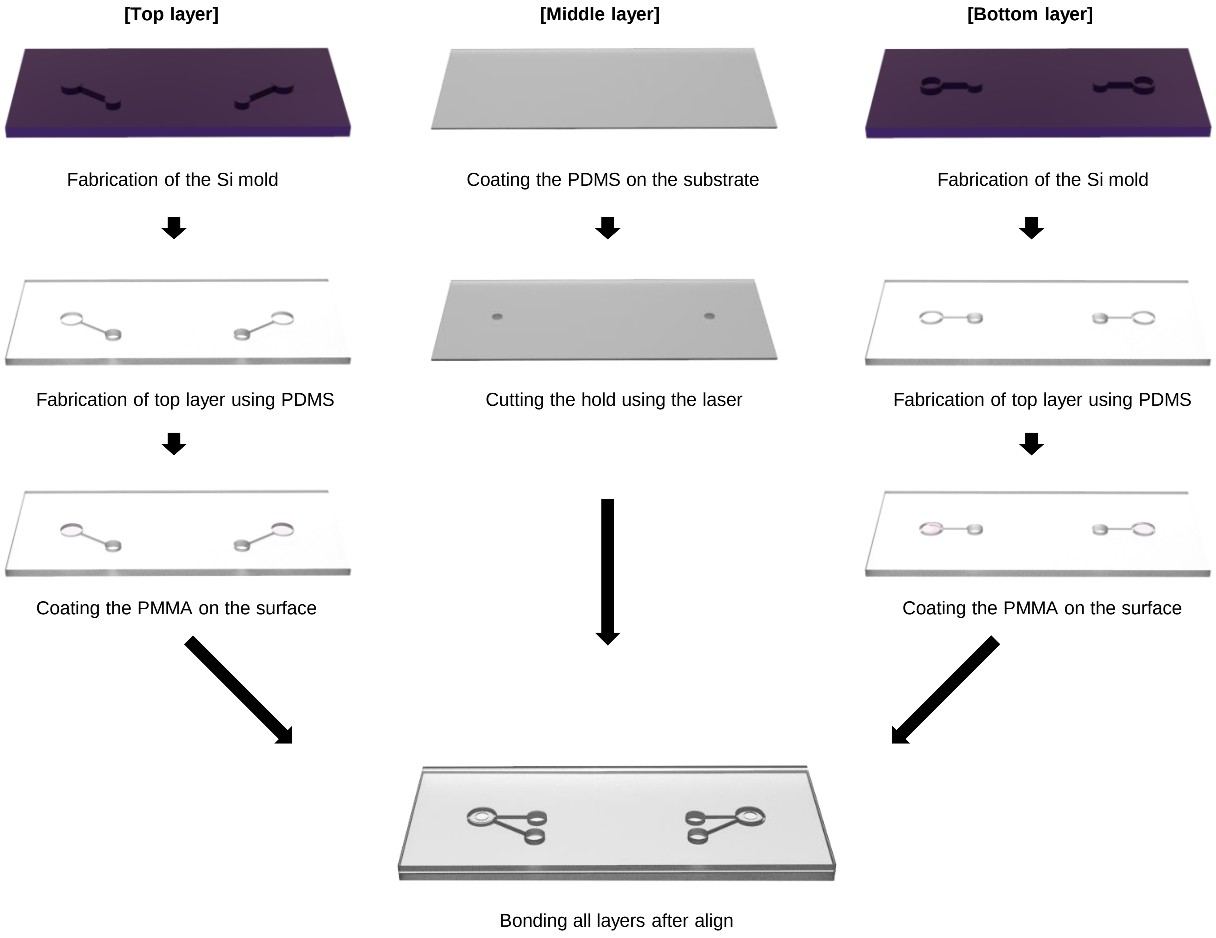

Supplementary Figure 10. Fabrication process of μ-fluidic flapper valve.

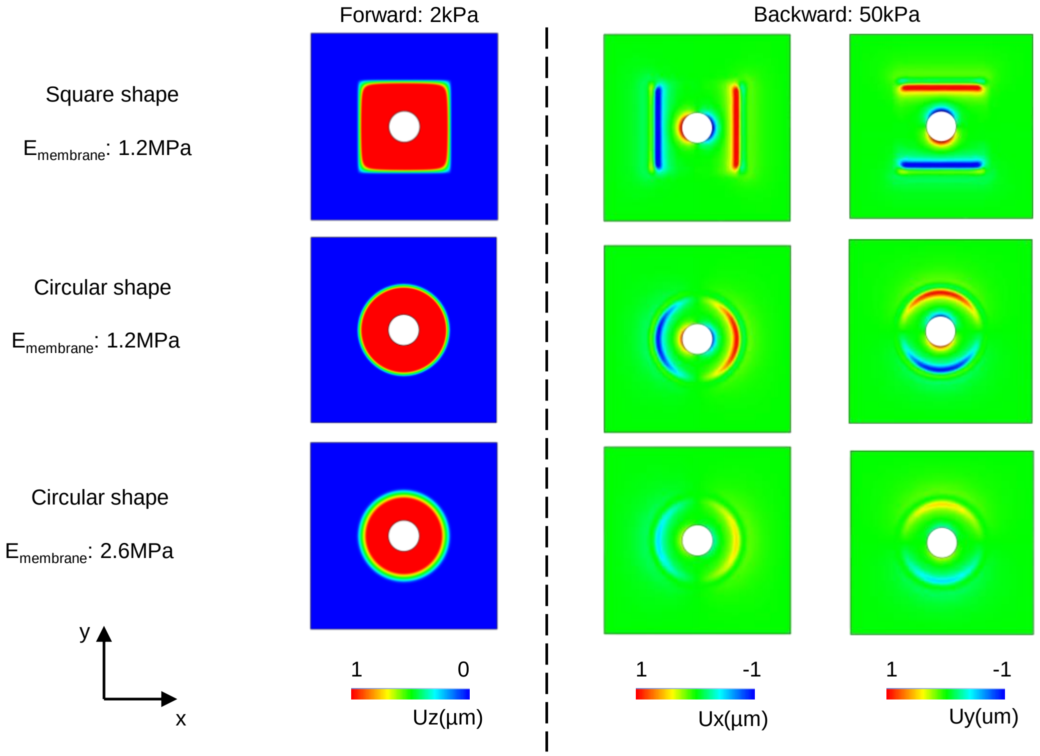

Supplementary Figure 11. Mechanical simulation of µ-fluidic flapper valve. Displacement of the flexible membrane along z direction(Uz) under 2 kPa forward pressure, and along x and y directions (Ux and Uy) under 50 kPa backward pressure has been simulated for 3 cases: 1) square step and 1:15 PDMS (Modulus: 1.2 Mpa), 2) circular step and 1:15 PDMS (Modulus: 1.2 Mpa), 3) circular step and 1:10 PDMS (Modulus: 2.6 Mpa).

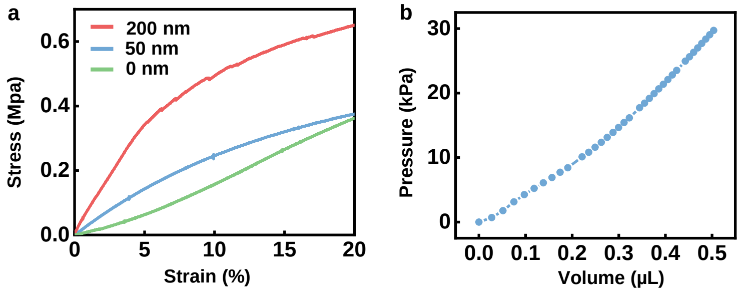

Supplementary Figure 12. Mechanical property of flexible membrane. (**a**) Strain-stress curves of SIS membrane with 0/50/200 nm gold coating. (**b**) Computational result of total pressure as a function of volume of drug delivered from the system. Source data are provided as a Source Data file.

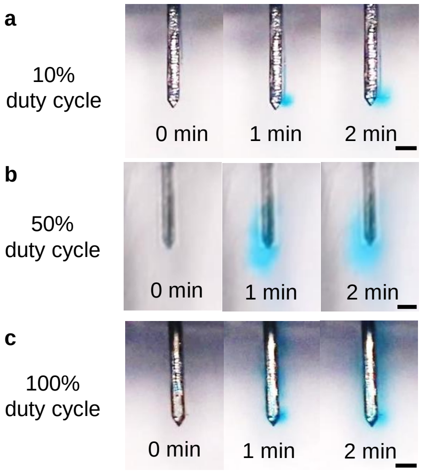

Supplementary Figure 13 Photos of the delivery of dyed aqueous solution in the hydrogel with optofluidic devices. The devices were operated at (**a**) 10% duty cycle, (**b**) 50% duty cycle, (**c**) 100% duty cycle, 250 ms frequency. Scale bar: 0.5 mm. The photo after 0, 1 and 2 min of delivery demonstrates that low duty cycle delivery allows for a lower flow rate, which can reduce the backflow effect.

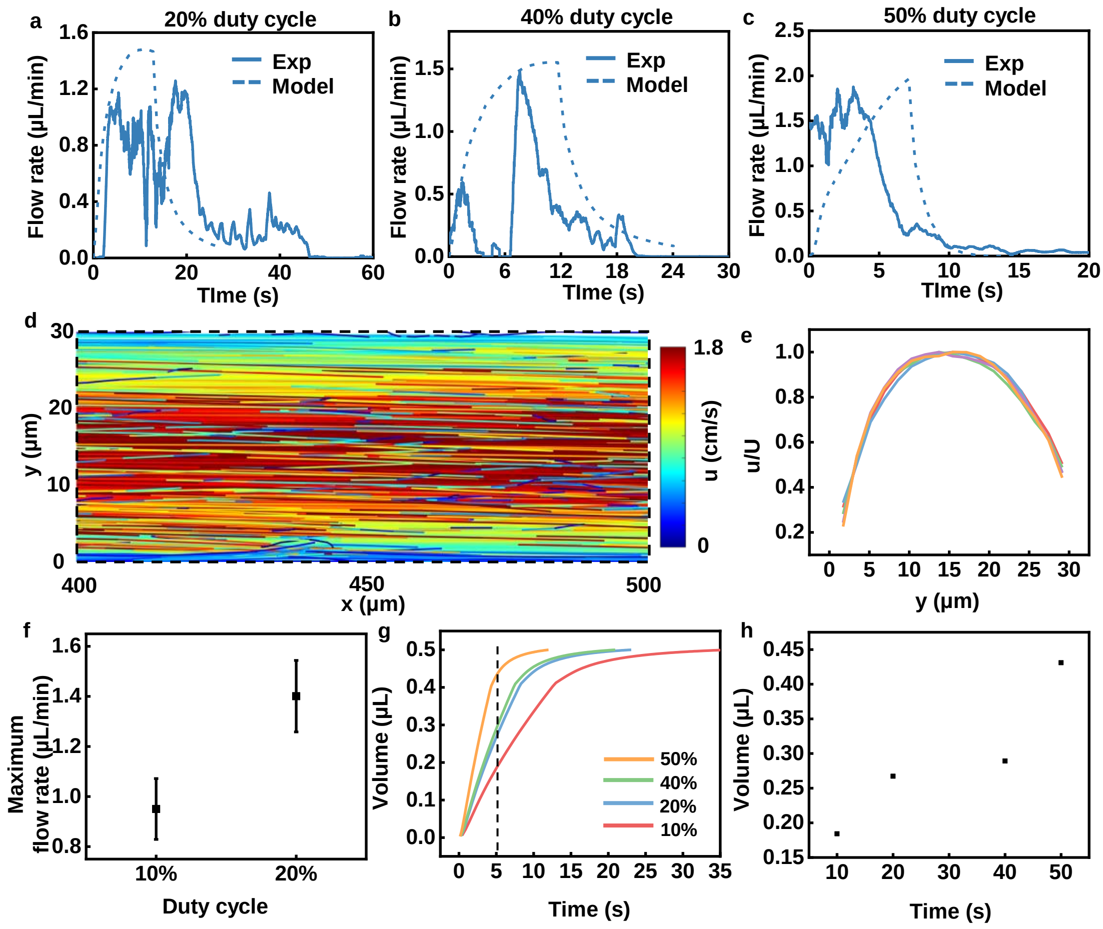

Supplementary Figure 14. Flow rate characteristics of the electrochemical μ-pump system. (**a**)-(**d**) Temporal profiles of the flow rate when operated at 250ms frequency and 20% duty cycle (**a**), 40% duty cycle (**b**), 50% duty cycle (**c**). (**d**) flow trajectories of (**Main Fig. 3g**) at 400 < x <500 um. (**e**) normalized velocity profiles at various streamwise locations. (**f**) Maximum flow rate at duty cycle 10% and 20%. Source data are provided as a Source Data file. n = 2 independent experiments. Data are presented as mean values +/- SEM.(**g**) Modeling result of delivered volume as a function of time, based on the effective current measured in the experiments. (**h**) Modeling result of delivered volume as a function of duty cycle at 5 seconds of delivery.

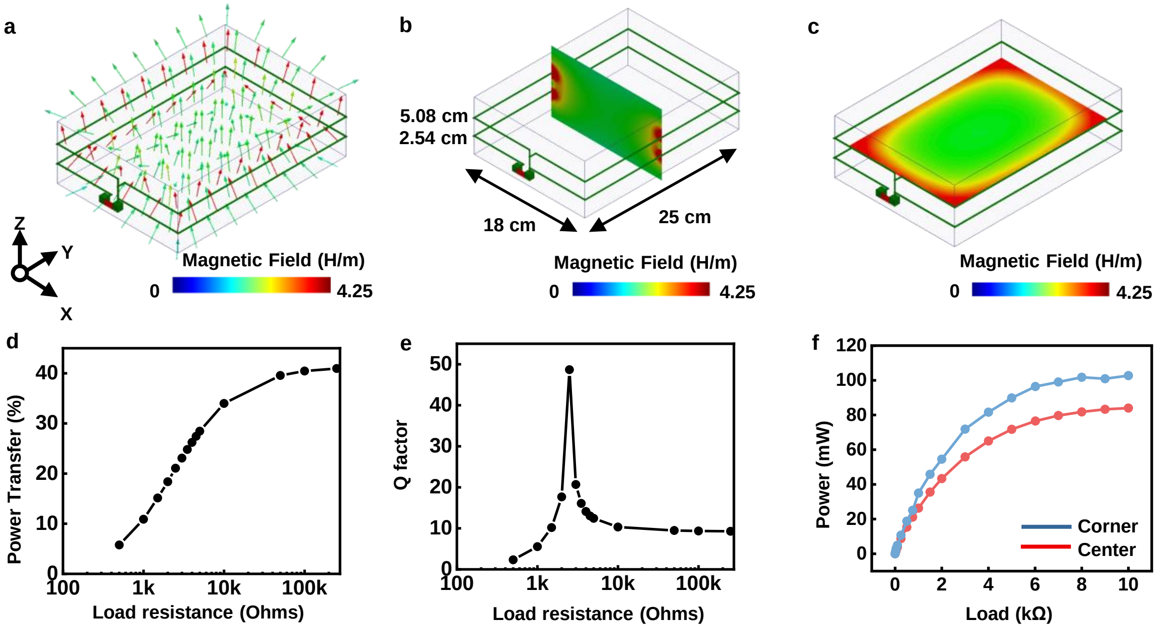

Supplementary Figure 15. Simulations and characterizations of primary and secondary antennas. (**a**)-(**c**). Computational result of magnetic field in the whole area (**a**) XY plane (**b**) and XZ plane (**c**) with transmitter antenna input power 8W. (**d**) Computational result of power transfer changing with load resistance. (**e**) Computational result of Q factor changes with load resistance. (**f**) Power harvested at corner and center of the cage over various loads. (8W) Source data are provided as a Source Data file.

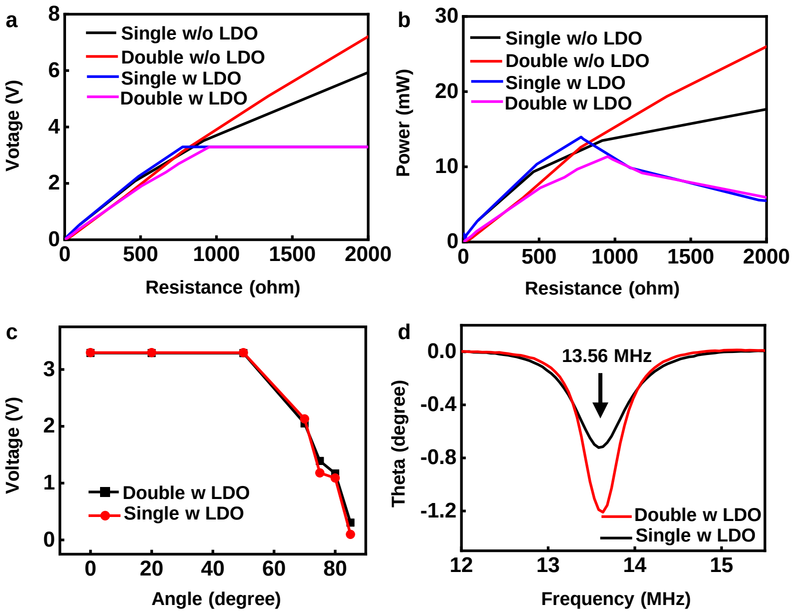

Supplementary Figure 16. Characteristics of single- and double- sided antenna. (**a**) Voltage change with load resistance for single- and double-sided antenna with/without LDO. (**b**) Power change with load resistance for single- and double-sided antenna with/without LDO. (**c**) Voltage change with tilted angle for single- and double-sided antenna with LDO. (**d**) Phase response of the receiver antenna for single- and double-sided antenna, demonstrating the resonant frequency at ~13.56 MHz. Single-sided: Q factor=17.372, FWHM=783278.72385. Double-sided: Q factor=24.512 f(peak)=1.36181*10^7, FWHM=555566.22192. Source data are provided as a Source Data file.

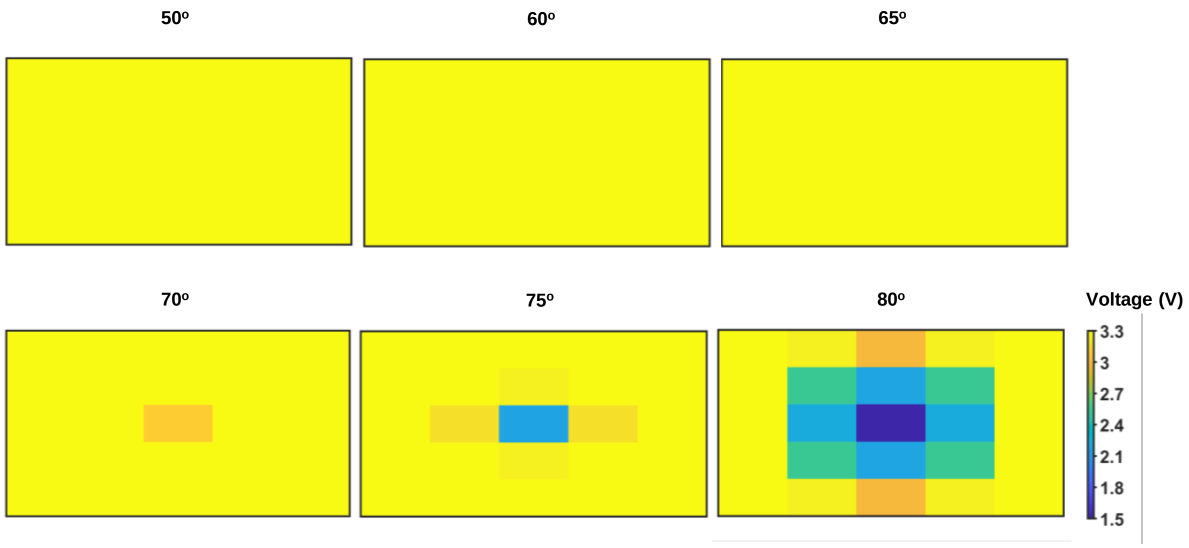

Supplementary Figure 17. Power distribution of the field at 1 inch with different angular misalignment (0 - 80º).

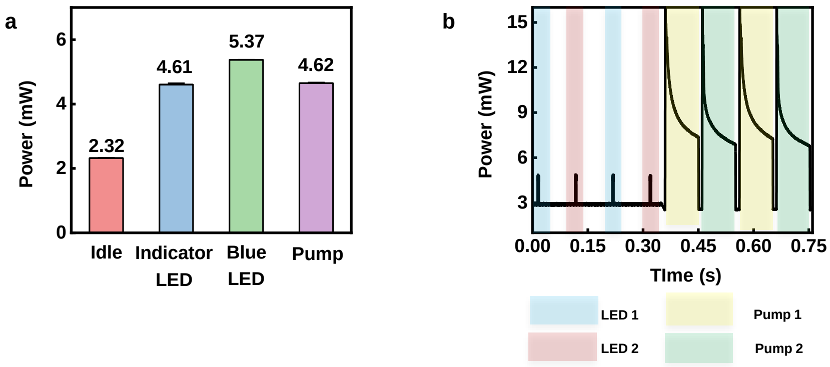

Supplementary Figure 18. Power consumption of optofluidic system. (**a**) Bar graph illustration of power consumption of four states individually. (**b**) Power consumption curves of out of phase operation for bilateral optical stimulation (frequency: 200 ms, duty cycle: 5%) and drug delivery (frequency 200 ms, duty cycle: 45%). Source data are provided as a Source Data file.

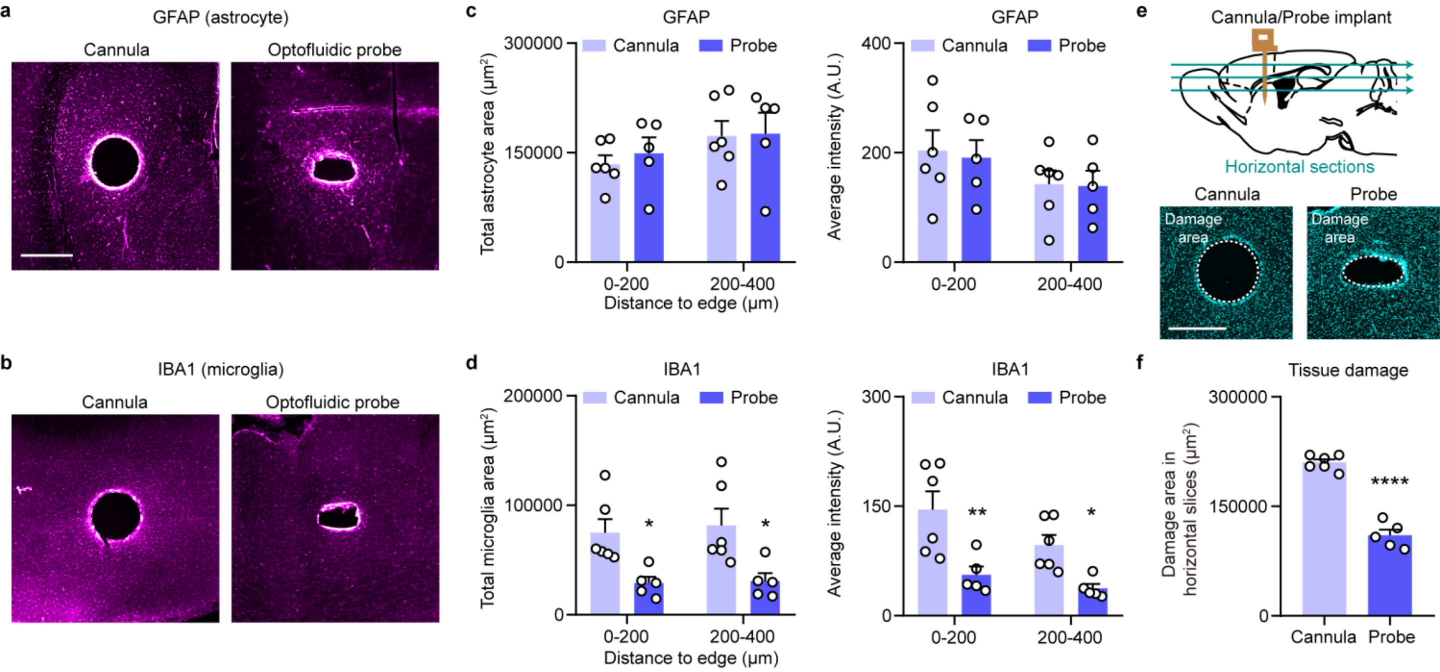

Supplementary Figure 19. Biocompatibility of injectable optofluidic probes

(**a**) Astrocytic (GFAP) immunoreactivity surrounding the implantation site of a commercial cannula (left) and an optofluidic probe (right). Scale bar: 500 µm. (**b**) Same as (**a**), but for microgila (IBA1). (**c**) Summary data showing total astrocyte dense (GFAP) area (Left) and average intensity of GFAP fluorescence (Right) at different distances from the edge of implantation. Two-way ANOVA, Sidak's multiple comparisons test (Cannula vs Probe), Area: 0-200 µm, $p = 0.8472$, 200-400 µm, $p = 0.9932$; intensity: 0-200 µm, $p = 0.9502$, 200-400 µm, $p = 0.9963$. $n = 6$ biologically independent samples in Cannula group, $n = 5$ biologically independent samples in Probe group. (**d**) Same as (**c**), but for microglia (IBA1). Two-way ANOVA, Sidak's multiple comparisons test (Cannula vs Probe), Area: 0-200 µm, $p = 0.0239$, 200-400 µm, $p = 0.0125$; intensity: 0-200 µm, $p = 0.0030$, 200-400 µm, $p = 0.0462$. $n = 6$ biologically independent samples in Cannula group, $n = 5$ biologically independent samples in Probe group.(**e**) Schematic (top) and example images (bottom) showing the measurement of damage area in cross sectional slices after implantation of cannula and probe. Scale bar: 500 µm. (**f**) Summary data show the size of damage area in horizontal brain slices. Two-tailed unpaired t-test, $p < 0.0001$. $n = 6$ biologically independent samples in Cannula group, $n = 5$ biologically independent samples in Probe group. Data represent mean +/- SEM, *$p < 0.05$, **$p < 0.01$, ****$p < 0.0001$. Source data are provided as a Source Data file.

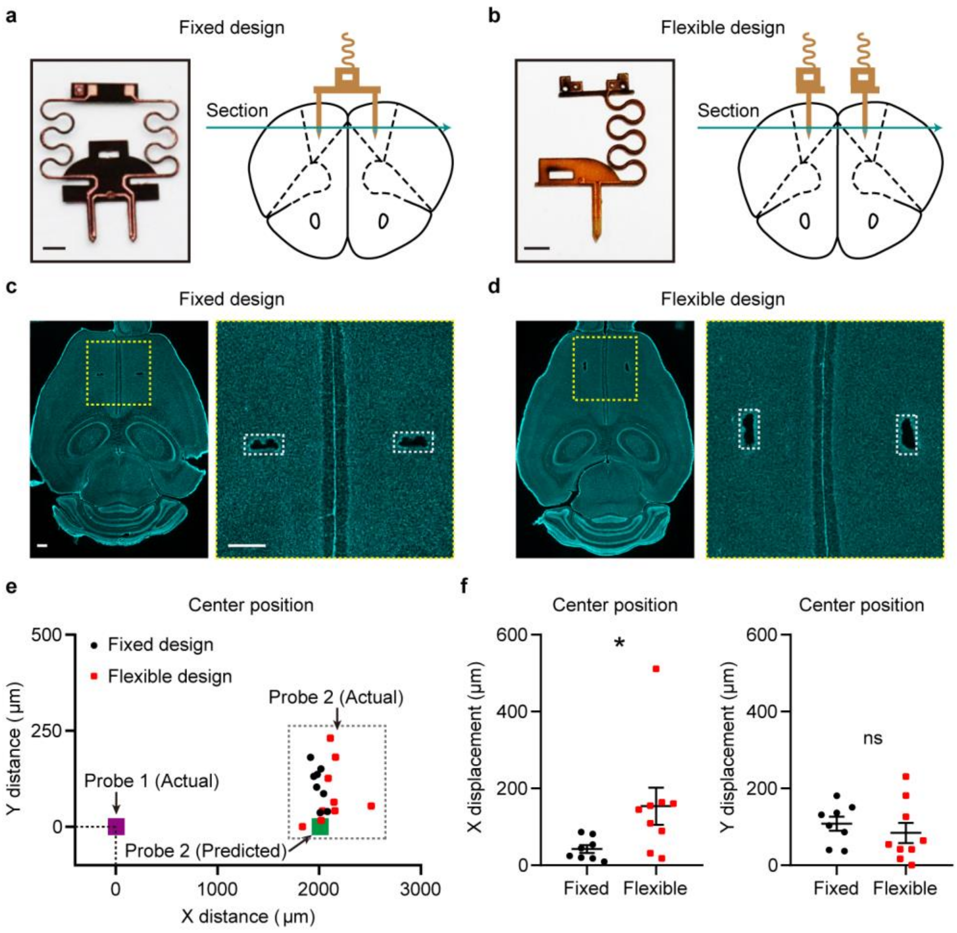

Supplementary Figure 20. Positioning accuracy of injectable optofluidic probes
(**a**) Image and schematic showing an integrated probe with two tips (fixed design) and subsequent histological assessment. Scale bar: 1 mm (**b**) Same as (**a**), but for two separately implantable probes (flexible design). (**c**) Example images showing a horizontal brain slice with the implantation locations of fixed design probes. Scale bar: 500 µm. (**d**) Same as (**c**), but for flexible design probes. (**e**) Scatter plot showing the distance between probes in X and Y axes for fixed and flexible probes. Purple square: actual position of probe 1. Green square: predicted position of probe 2. n = 8 biologically independent samples in Fixed group, n = 9 biologically independent samples in Flexible group. (**f**) Left, summary data showing the displacement in X axis between probes for fixed and flexible designs. Two-tailed unpaired t-test, $p = 0.0498$. Right, same as left, but for Y axis. Two-tailed unpaired t-test, $p = 0.4744$. n = 8 biologically independent samples in Fixed group, n = 9 biologically independent samples in Flexible group. Data represent mean +/- SEM, *$p < 0.05$. Source data are provided as a Source Data file.

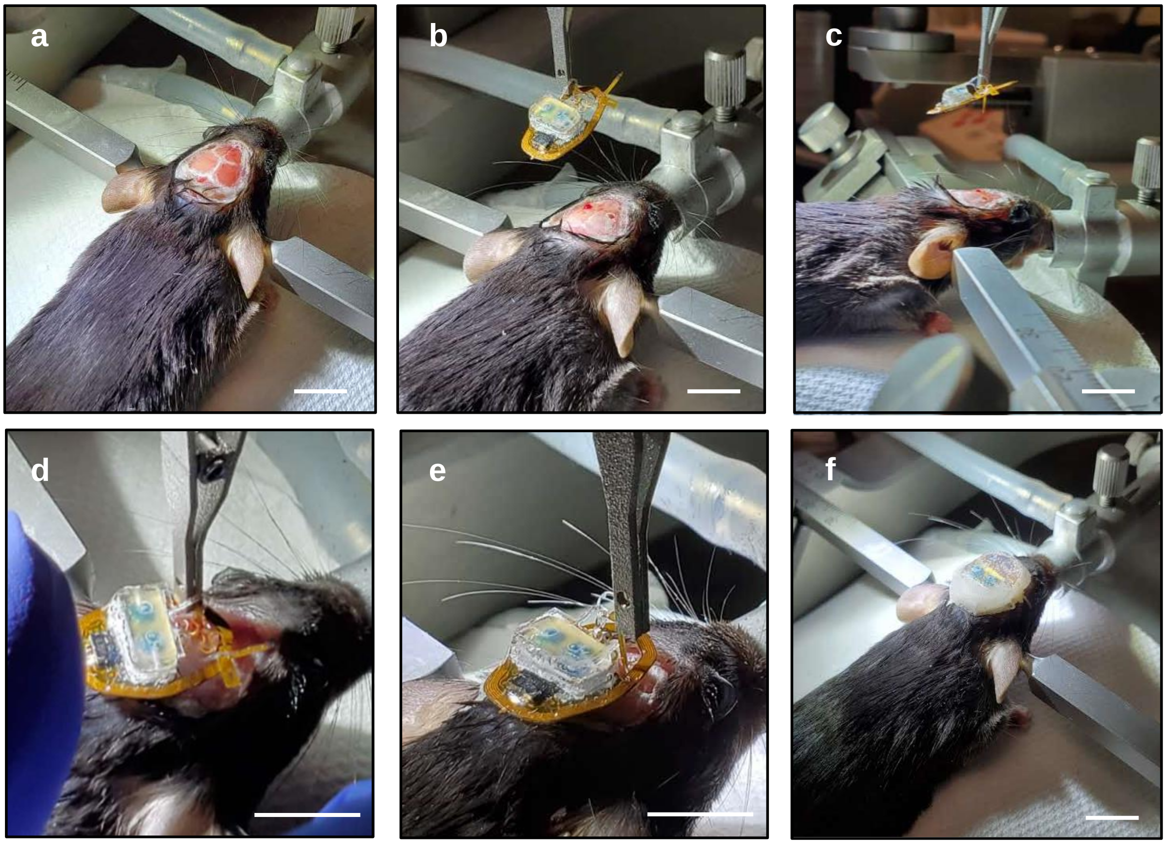

Supplementary Figure 21. Illustration of surgical procedures for implanting the bilateral optofluidic device. (**a**) Preparation for implantation by exposing the skull. (**b**) and (**c**) Positioning the device. (**d**) Implantation of the left tip. (**e**) Implantation of the right tip. (**f**) Stabilization of the device by dental cement. Scale: 1 cm.

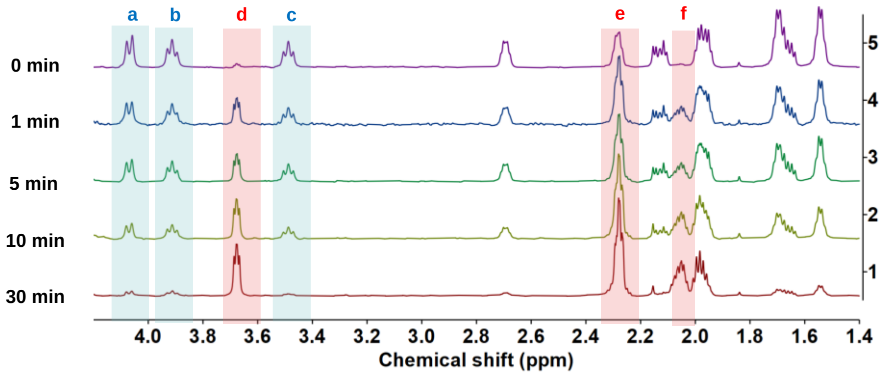

Supplementary Figure 22. NMR spectrum of rubi-glutamate with 0, 1, 5, 10, 30mins blue μ-LED irradiation. The signals a-c correspond to the coordinated amine protons of rubi-glutamate, while d-f correspond to free glutamate. Source data are provided as a Source Data file.
