# Supplementary material for: Wireless multi-lateral optofluidic microsystems for real-time programmable optogenetics and photopharmacology
Source: Nat Commun. 2022 Sep 22;13:5571. doi: 10.1038/s41467-022-32947-0 (PMC9500026; doi:10.1038/s41467-022-32947-0)
Supplement: Supplementary file 3 — Reporting Summary [file 41467_2022_32947_MOESM3_ESM.pdf]

nature portfolio

Corresponding author(s): Yonggang Huang, Yevgenia Kozorovitskiy, John A. Rogers

Last updated by author(s): Jul 30, 2022

Reporting Summary

Nature Portfolio wishes to improve the reproducibility of the work that we publish. This form provides structure for consistency and transparency in reporting. For further information on Nature Portfolio policies, see our Editorial Policies and the Editorial Policy Checklist.

Statistics

For all statistical analyses, confirm that the following items are present in the figure legend, table legend, main text, or Methods section.

n/a	Confirmed	
☐	☒	The exact sample size (*n*) for each experimental group/condition, given as a discrete number and unit of measurement
☐	☒	A statement on whether measurements were taken from distinct samples or whether the same sample was measured repeatedly
☐	☒	The statistical test(s) used AND whether they are one- or two-sided<br>*Only common tests should be described solely by name; describe more complex techniques in the Methods section.*
☐	☒	A description of all covariates tested
☐	☒	A description of any assumptions or corrections, such as tests of normality and adjustment for multiple comparisons
☐	☒	A full description of the statistical parameters including central tendency (e.g. means) or other basic estimates (e.g. regression coefficient) AND variation (e.g. standard deviation) or associated estimates of uncertainty (e.g. confidence intervals)
☐	☒	For null hypothesis testing, the test statistic (e.g. *F*, *t*, *r*) with confidence intervals, effect sizes, degrees of freedom and *P* value noted<br>*Give P values as exact values whenever suitable.*
☒	☐	For Bayesian analysis, information on the choice of priors and Markov chain Monte Carlo settings
☒	☐	For hierarchical and complex designs, identification of the appropriate level for tests and full reporting of outcomes
☒	☐	Estimates of effect sizes (e.g. Cohen's *d*, Pearson's *r*), indicating how they were calculated

Our web collection on statistics for biologists contains articles on many of the points above.

Software and code

Policy information about availability of computer code

Data collection	Mediso Nucline v2.01 was used to collect MicroCT imaging data. Raspberry Pi Camera v2 (Raspberry Pi Foundation) was used for video acquisition in behavioral experiments.
Data analysis	Group statistical analyses were done using GraphPad Prism 7 software (GraphPad, LaJolla, CA).<br>All image data were analyzed by using FIJI (ImageJ 1.52p) (Schindelin et al., 2012).<br>Estimation of mouse trajectories in rotational behaviors was done with Deeplabcut (2.1) (Mathis et al, 2018) and Python 3.9 (Python Software Foundation).<br>Plots were generated in OriginPro2019 and GraphPad Prism 7 software (GraphPad, LaJolla, CA).<br>MicroCT imaging data were analyzed with Amira v2020.2.<br>Mechanical structural simulation data were generated in ABAQUS (2016).<br>Electromagnetic analysis were performed in ANSYS HFSS 2020 Electronic Desktop.<br>Nuclear Magnetic Resonance spectrum was generated by MNOVA v.14 (Mestrelab Research, Spain).<br>All computer code generated during and/or used in the current study is available available at https://github.com/A-VazquezGuardado/Smart_NFC_Optofluidics.

For manuscripts utilizing custom algorithms or software that are central to the research but not yet described in published literature, software must be made available to editors and reviewers. We strongly encourage code deposition in a community repository (e.g. GitHub). See the Nature Portfolio guidelines for submitting code & software for further information.

Data

Policy information about availability of data

All manuscripts must include a data availability statement. This statement should provide the following information, where applicable:

- Accession codes, unique identifiers, or web links for publicly available datasets
- A description of any restrictions on data availability
- For clinical datasets or third party data, please ensure that the statement adheres to our policy

Raw video data generated during the current study are available from the corresponding author on reasonable request. The numerical data generated and analyzed in the current study are available available at https://github.com/A-VazquezGuardado/Smart_NFC_Optofluidics.

Human research participants

Policy information about studies involving human research participants and Sex and Gender in Research.

Reporting on sex and gender	No human participants are involved in this study.
Population characteristics	No human participants are involved in this study.
Recruitment	No human participants are involved in this study.
Ethics oversight	No human participants are involved in this study.

Note that full information on the approval of the study protocol must also be provided in the manuscript.

Field-specific reporting

Please select the one below that is the best fit for your research. If you are not sure, read the appropriate sections before making your selection.

☒ Life sciences ☐ Behavioural & social sciences ☐ Ecological, evolutionary & environmental sciences

For a reference copy of the document with all sections, see nature.com/documents/nr-reporting-summary-flat.pdf

Life sciences study design

All studies must disclose on these points even when the disclosure is negative.

Sample size	Required sample sizes were estimated based on previous report of published results and our past experience performing similar experiments. No statistical methods were used to pre-determine sample sizes but our sample sizes are similar to those reported in previous publications (Shin et al, 2017; Kingsbury et al, 2019; Wu et al, 2021; Yang et al, 2021)
Data exclusions	Data from failed devices were excluded from the analysis.
Replication	The number of replicates for each experiment were reported in the paper and the method section. All representative data were from experiments that were independently repeated for at least five times with similar results. All attempts at replication were successful.
Randomization	Animals were randomly assigned to treatment groups. All samples were randomly assigned to experimental groups.
Blinding	All behavioral experiments were analyzed blind to conditions. The investigators were blinded to group assignment during data collection. Blinding was not relevant to other work since the metrics were objectively quantified and analyzed.

Reporting for specific materials, systems and methods

We require information from authors about some types of materials, experimental systems and methods used in many studies. Here, indicate whether each material, system or method listed is relevant to your study. If you are not sure if a list item applies to your research, read the appropriate section before selecting a response.

Materials & experimental systems

n/a	Involved in the study	
☐	☒	Antibodies
☒	☐	Eukaryotic cell lines
☒	☐	Palaeontology and archaeology
☐	☒	Animals and other organisms
☒	☐	Clinical data
☒	☐	Dual use research of concern

Methods

n/a	Involved in the study	
☒	☐	ChIP-seq
☒	☐	Flow cytometry
☒	☐	MRI-based neuroimaging

Antibodies

Antibodies used

For evaluation of biocompatibility, rabbit polyclonal anti-GFAP (1:1000, ab7260, abcam, Cambridge, United Kingdom), and rabbit monoclonal anti-IBA1 (1:1000, ab178846, abcam) were used. Alexa Fluor 647-conjugated secondary antibodies against rabbit (Life Technologies, Carlsbad, CA) were diluted at 1:500.

Validation

All antibodies are commercially available and validated in multiple studies. Complete information is available in the data sheets on the manufacturer's website. According to the manufacturer's website, rabbit anti-GFAP specifically recognizes mammalian GFAP on western blots and immunocytochemically. It detects a band of 55kDa corresponding to GFAP and also a GFAP derived 48kDa band. Rabbit anti-IBA1 is a rabbit monoclonal antibody generated with synthetic peptide within Mouse Iba1 aa 100 to the C-terminus. The recognition specificity has been validated in multiple mouse, rat, and human cell lines and tissues according to the manufacturer's website (https://www.abcam.com/iba1-antibody-epr16588-ab178846.html).

Animals and other research organisms

Policy information about studies involving animals; ARRIVE guidelines recommended for reporting animal research, and Sex and Gender in Research

Laboratory animals

All experiments used young adult wild-type C57BL/6J mice or hybrid C57BL/6J mice on Sv129 background (>8 weeks old and 20-30 g at start of experiments; Jackson Labs and Charles River), maintained at ~25°C and humidity ranged of 30% to 70%. Mice were maintained on a 12-h light/dark cycle (lights on at 6:00 – 7:00 AM) and fed ad libitum.

Wild animals

No wild animals were used in the study.

Reporting on sex

Approximately equal numbers of males and females were used for all experiments.

Field-collected samples

No field collected samples were used in the study.

Ethics oversight

Animals were handled according to protocols approved by the Animal Care and Use Committees in Northwestern University.

Note that full information on the approval of the study protocol must also be provided in the manuscript.
