# Supplementary material for: Wireless multi-lateral optofluidic microsystems for real-time programmable optogenetics and photopharmacology
Source: Nat Commun. 2022 Sep 22;13:5571. doi: 10.1038/s41467-022-32947-0 (PMC9500026; doi:10.1038/s41467-022-32947-0)
Supplement: Supplementary file 4 — Supplementary Files [file 41467_2022_32947_MOESM4_ESM.pdf]

Description of Additional Supplementary Files

Supplementary Video 1. Real-time Selective Control of Independent Subject Demonstrated on Plastic Mouse Models

Supplementary Video 2. Bilateral Optogenetic Control of Motor Behavior
